# Nutritional Composition, Health Benefits and Claim Conditions of Fish from Aquaculture: A Narrative Review

**DOI:** 10.3390/nu18081270

**Published:** 2026-04-17

**Authors:** Hanna Skendrović, Greta Krešić, Snježana Zrnčić, Jelka Pleadin

**Affiliations:** 1 Laboratory for Analytical Chemistry, Croatian Veterinary Institute, Savska Cesta 143, 10000 Zagreb, Croatia; skendrovic@veinst.hr; 2 Faculty of Tourism and Hospitality Management, University of Rijeka, Primorska 42, P.O. Box 97, 51410 Opatija, Croatia; gretak@fthm.hr; 3 Laboratory for Aquatic Animal Diseases, Croatian Veterinary Institute, Savska Cesta 143, 10000 Zagreb, Croatia; zrncic@veinst.hr

**Keywords:** nutrition claim, health claim, fish, health, sea bream, sea bass

## Abstract

This review addresses the nutritional composition, health benefits, and claim conditions of aquaculture fish, focusing on gilthead sea bream (*Sparus aurata*) and European sea bass (*Dicentrarchus labrax*). Both species provide high-quality proteins, essential amino acids, and favorable lipid profiles, particularly long-chain omega-3 fatty acids, alongside minerals such as phosphorus and selenium, which meet EU criteria for several authorized nutrition and health claims. Evidence demonstrates that regular consumption supports cardiovascular, cognitive, and visual health, reduces inflammation, and contributes to better pregnancy and early childhood outcomes. Consumer skepticism toward aquaculture persists, often driven by perceptions of reduced nutritional quality, despite evidence that farmed fish provide nutritionally valuable proteins and beneficial lipids. Nevertheless, both species consistently meet the requirements for multiple nutrition and health claims, particularly those related to protein, omega-3 fatty acids, and selected minerals, allowing their effective use in labeling and consumer communication. Clear, evidence-based labeling of such claims is crucial to enhance acceptance and promote farmed fish as safe, sustainable, and health-promoting dietary choices.

## 1. Introduction

Aquaculture, as the fastest growing food sector, is expected to play an increasing role in meeting the world’s rising demand for fish products [1,2,3,4,5]. With its rapid expansion, the global aquaculture industry has already surpassed wild-caught seafood production [6,7]. According to the most recent report [8], the rising consumption and commercialization of aquaculture fish have been accompanied by increased attention to their quality, nutritional value, and consumer acceptance [3].

Two major marine finfish species reared in the Mediterranean region are European sea bass (*Dicentrarchus labrax*, Linnaeus 1758) and gilthead sea bream (*Sparus aurata*, Linnaeus 1758), both of significant commercial value, which began to increase in the late 20th century [2,9,10,11,12]. European sea bass production in 2020 was approximately 244,000 tonnes, and sea bream production in 2022 was 564,000 tonnes [2,8]. These species are widely valued by consumers for their delicate flavour, white flesh, and relatively low fat content [9]. Nearly 90% of the world’s supply of sea bream and sea bass comes from just six countries, with Turkey contributing the largest share (37%), followed by Greece (25%), Egypt and Spain (14% and 10%), while Tunisia and Italy each account for approximately 4% of total output [13]. Consumption patterns of these two commercially important species vary across regions, with Greece, Portugal, and Cyprus having the highest per capita sea bass consumption in 2016: 796 g, 680 g, and 643 g respectively, followed by Spain and Italy (545 g and 513 g) [14].

With aquaculture now supplying nearly half of global fish consumption, understanding the nutritional value of farmed species, with particular emphasis on sea bass and sea bream, is critical for public health and sustainable food systems [2,3,15]. The quality and nutritional composition of farmed fish are influenced by several factors, including seasonal and biological differences (species, size, age, and sex), food source, health, and environmental factors (salinity, temperature, and contaminants) [16,17]. For example, sea bream has a high salinity tolerance, making it more valuable in the aquaculture sector [8,11].

Consumers with greater knowledge about fish tend to consume it more frequently, recognising the health benefits of omega-3 (*n*-3) fatty acids and high-quality proteins, particularly in the prevention of cardiovascular and neurological diseases [18,19]. Health and nutritional motivations positively impact fish intake, increasing the likelihood of white fish consumption by 2.8% and fatty fish by 2.5% [20]. However, consumption patterns vary regionally, depending on economic, cultural, and geographic factors [21].

Although fish is predominantly perceived as a healthy food, farmed fish is often seen as less natural, potentially unhealthy, and suspected of containing undesirable substances such as antibiotics [18]. Consumer perception studies indicate that aquaculture is not necessarily viewed negatively, although consumers generally prefer wild fish, which they associate with superior quality, taste, nutritional value and safety [19,21]. Therefore, informing consumers about the nutritional and health benefits of farmed fish is crucial, as misconceptions may lead them to avoid it [19].

Various claims on food can serve as communication tools, potentially encouraging consumers to choose products they might otherwise approach with scepticism, such as farmed fish. According to European Regulation [22], a claim is defined as any message, symbol, or representation, including pictorial or graphic form, that states, suggests, or implies that a food has specific characteristics. Nutrition and health claims [23,24] are the two main categories of claims that represent the focus of this paper, while within the EU regulatory framework their use on fish products must be scientifically substantiated to protect consumers [22,23,24,25].

This narrative review provides a comprehensive overview of the scientific literature on the nutritional composition of fish from aquaculture, the evidence-based health benefits associated with its consumption, and the conditions of use for nutrition and health claims, with special emphasis on sea bass and sea bream as the most important economic aquaculture fish species [26].

The remainder of the manuscript is structured as follows: Section 2 describes the literature search methodology, Section 3 outlines the nutritional composition of aquaculture fish, Section 4 discusses the health benefits associated with fish consumption, and Section 5 presents the regulatory criteria for nutrition and health claims, along with a comparative evaluation of gilthead sea bream and European sea bass in this context.

## 2. Literature Search

A comprehensive literature search was conducted to identify studies on the nutritional composition, health benefits, and authorized nutrition and health claims of gilthead sea bream (*Sparus aurata*) and European sea bass (*Dicentrarchus labrax*). The following electronic databases were searched: Web of Science, Scopus, Elsevier, PubMed, and Google Scholar. Key search terms included “gilthead sea bream,” “Sparus aurata,” “European sea bass,” “Dicentrarchus labrax,” “nutrition claims,” “health claims,” and “chemical analysis.” Studies were screened for relevance based on their focus on the chemical composition of these species, nutrient content, or the applicability of authorized nutrition and health claims. Only studies published in the last 20 years, written in English, and providing original or review data on these fish species were considered. This approach ensured a comprehensive overview while following principles inspired by PRISMA guidelines for transparent reporting of literature selection.

The nutritional ranges from the literature sources identified through the search process are presented in the Section 5. Table 1 and Table 2 summarize the reported ranges of nutrient composition for gilthead sea bream, while Table 3 and Table 4 present the corresponding data for European sea bass. The values represent aggregated ranges reported across the selected studies.

## 3. Nutritional Composition of Fish from Aquaculture

The valuable nutritional composition of fish derives not only from its low energy value, but also from its high nutrient density, due to essential macronutrients such as high-quality protein and fat (long-chain polyunsaturated fatty acids (LC-PUFAs)—mainly eicosapentaenoic acid (EPA) and docosahexaenoic acid (DHA)), as well as micronutrients such as vitamins (D, A, and B12) and minerals (iodine, selenium, and iron) [4,16,27,28,29].

Due to a complete matrix of macronutrients and micronutrients that support human health throughout the life cycle, inclusion of fish in the diet is not only beneficial but also essential in regions where nutritional deficiencies are prevalent [8,9,15,30,31]. Its consumption is of particular importance in developing countries, where fish can represent a major source of animal protein, with some reports suggesting that it may contribute substantially to daily animal protein intake in certain populations [8,15,31].

### 3.1. Macronutrients

Considering their prevalence, quality, and importance for human nutrition, fish provide an excellent source of high-quality proteins, essential amino acids, and health-promoting lipids [15,16,17,31,32,33]. They supply 12–25% highly digestible protein containing all essential amino acids in proportions that meet human dietary needs, making them nutritionally superior to many terrestrial animal proteins such as milk, eggs, and meat [12,16,17,30,31]. Compared to terrestrial meats, fish protein is richer in amino acids such as lysine, methionine, cysteine, and taurine, and includes bioactive peptides with demonstrated physiological effects [31]. These proteins and peptides play structural roles in skeletal muscle, and also support growth, digestive function, tissue repair, enzymatic and hormonal activity, and overall metabolic regulation [12,15,30,34]. Taurine, abundant in fish, contributes to osmoregulation, bile salt synthesis, and neural development [34].

Aquatic animal foods could be classified as lean protein sources, since this food group poses a lower caloric density and higher protein content compared with terrestrial animals [31,34]. Fish and seafood represent the third most important source of animal protein worldwide, after cereals and milk, and contribute around 17.1% to total animal protein intake [2,34].

Fish lipid content varies widely, from 0.2% to 30%, allowing categorisation into lean (<2.5%), semi-fatty (2.5–6%), and fatty fish (>6%), with gilthead sea bream and sea bass classified as semi-fatty [15,16,17]. Polyunsaturated fatty acids (PUFAs), particularly *n*-3s, are key components of fish fat, essential for cardiovascular health and neurological development in children, and are abundant in both lean and fatty species [15,16]. Fish also contribute minimally to dietary cholesterol intake, with average values around 35 mg per 100 g [16].

Although carbohydrates are nearly absent in fish (<0.5%), glycogen is present in trace amounts, primarily serving as short-term energy storage [12,16].

### 3.2. Fatty Acids

Fatty acids are among the most valuable nutrients in fish, playing a vital role in human health and disease prevention [35]. Fish lipids include saturated fatty acids (SFAs), monounsaturated fatty acids (MUFAs), and PUFAs, including essential *n*-3 and omega-6 (*n*-6) fatty acids, which must be obtained through the diet as the human body cannot synthesise them endogenously [30,31]. Marine-derived foods typically have low SFA content, and its regular consumption aligns with recommendations to reduce dietary SFA intake to lower the risk of cardiovascular diseases (CVD) [30]. PUFAs are straight-chain fatty acids with two or more double bonds and carbon chains ranging from 18 to 22 atoms [36,37]. Classification of these fatty acids into *n*-3 and *n*-6 families is based on the position of the first double bond, with the *n*-3 group including alpha-linolenic acid (ALA), EPA, docosapentaenoic acid (DPA), and DHA, while the *n*-6 group consists of linoleic acid (LA) and arachidonic acid (AA) [37].

Fish are a unique and essential dietary source of *n*-3 LC-PUFAs, particularly EPA and DHA, which are crucial for health from fetal development through ageing [16,27,31,38]. As the primary dietary sources of EPA and DHA, fish and seafood contain varying concentrations depending on species, geographic origin, and seasonality [38,39]. EPA and DHA are particularly abundant in marine fish such as gilthead sea bream and European sea bass, providing approximately 0.1–0.3 g per adult serving, usually with more EPA than DHA [36,40].

These fatty acids are incorporated into phospholipids in cell membranes, where they regulate membrane fluidity, intracellular signalling, and gene expression related to lipid metabolism and inflammation [41]. Beyond their structural role, EPA and DHA have significant regulatory effects on key physiological processes, particularly those related to inflammation, lipid homeostasis, and vascular function [39]. They serve as precursors to lipid mediators such as eicosanoids, resolvins, and protectins, which contribute to vasodilation, immune modulation, thrombogenesis, and the resolution of inflammation. As a result, they exert a broad range of biological effects, including anti-inflammatory, antithrombotic, antiarrhythmic, antioxidant, anticancer, antiadipogenic, and neuroprotective actions [32,34,39,41].

**Figure 1:**
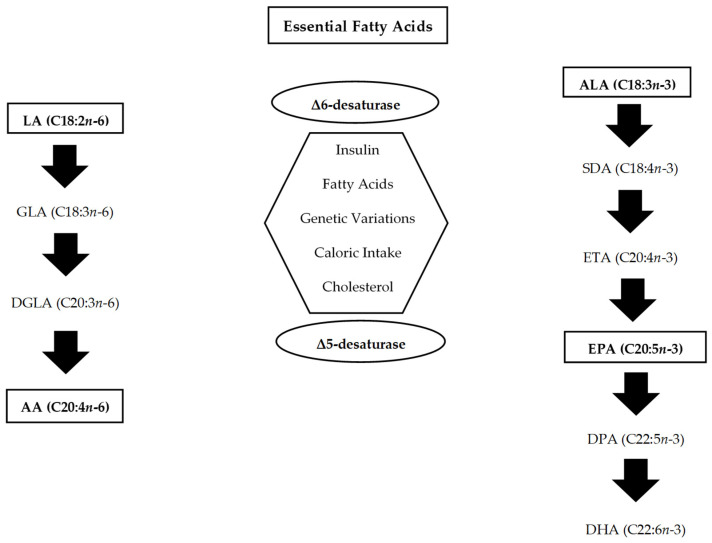
Bioconversion of PUFAs [37]. LA—linoleic acid; GLA—gamma-linoleic acid; DGLA—dihomo-gamma-linoleic acid; AA—arachidonic acid; ALA—alpha-linolenic acid; SDA—stearidonic acid; ETA—eicosatetraenoic acid; EPA—eicosapentaenoic acid; DPA—docosapentaenoic acid; DHA—docosahexaenoic acid.

LC-PUFAs are synthesised from the fish-derived essential fatty acids LA (18:2 *n*-6) and ALA (18:3 *n*-3), which act as necessary metabolic precursors [17,36,37,42]. However, the metabolic conversion of ALA to long-chain *n*-3 fatty acids in humans is limited, with EPA synthesised at roughly 3% efficiency and DHA at approximately 1.9% (Figure 1) [1,17,37,43]. This process involves enzymatic cascades of desaturation, elongation, and oxidation, but it is insufficient to meet physiological requirements [1,37].

The enzymatic conversion of dietary ALA into EPA and DHA depends on various physiological and genetic factors, such as age, sex, health status, and hormonal influences [36]. Studies suggest that women generally exhibit higher DHA concentrations in the bloodstream than men, likely due to more efficient enzymatic conversion of ALA to DHA, potentially influenced by hormonal factors [44]. Although certain adaptations exist, endogenous synthesis in humans is insufficient to maintain optimal tissue concentrations, highlighting the need for marine sources such as fish and algae [1,37].

Numerous studies have indicated that consistent consumption of EPA and DHA lowers the risk of CVD, cancer, Alzheimer’s disease, cognitive disorders, macular degeneration, rheumatoid arthritis, and various chronic illnesses [30,34]. Their pleiotropic functions in regulating inflammation and metabolic health underpin their importance in preventing disorders such as diabetes, obesity, stroke, and neurodegeneration [32,33,41]. Although their benefits are established, further studies are needed to better understand their individual physiological roles and clinical implications [45].

Although gilthead sea bream and European sea bass have limited enzymatic ability to synthesize LC-PUFAs, their low desaturase and elongase activity necessitates the intake of preformed EPA and DHA through diet or enriched feeds [5,17]. Considerable amounts of EPA (1.83–1.91 g/100 g) and DHA (3.51–3.98 g/100 g) have been identified in sea bream viscera. Nevertheless, these values remain below those observed in oily fish species such as tuna and salmon [17].

Multiple variables, such as fish species, body size, age, sex, feed type, water temperature, salinity, and aquaculture conditions, can influence the composition of lipids in fish tissue [15,16,17]. Temperature fluctuations have been shown to alter the balance between SFAs and MUFAs, thereby affecting lipid biosynthesis pathways in fish [46]. According to [47], exposure to cooler water conditions results in elevated concentrations of *n*-3 LC-PUFAs in various tissues of sea bass.

Numerous authoritative health organisations, including the Food and Agriculture Organization (FAO), the World Health Organization (WHO), the American Heart Association (AHA), and the European Food Safety Authority (EFSA), recommend regular consumption of fish to meet EPA and DHA requirements essential for the primary and secondary prevention of CVD [29,30,45,48,49]. The general recommendation is 250 to 500 mg of EPA + DHA per day, typically provided through two to three weekly servings of fish; individuals with heart disease are advised to increase this to 1 g per day, while those with high triglycerides may benefit from 2 to 4 g daily [4,30,33,48,49,50]. These intakes are generally achievable by consuming one to two servings of fatty fish per week [30,31,35,39].

### 3.3. Micronutrients: Vitamins and Minerals

Fish are a vital source of vitamins, particularly fat-soluble vitamins A, D, and E, as well as water-soluble B-complex vitamins such as thiamine, riboflavin, niacin, and cobalamin [15,16,30]. The concentrations of micronutrients, including vitamins and minerals, vary among fish species and are influenced by seasonal variation and dietary intake [16]. The ranges of selected vitamins and minerals in sea bream (Table 1) and sea bass (Table 3) are provided in the respective tables; the following text discusses their nutritional relevance without reiterating the specific values.

Vitamin A, a fat-soluble vitamin from fish, poses high bioavailability and supports vision and bone growth [16]. It also plays an important role in immune function, reproduction, and cell differentiation [34]. Fish liver and oil are important natural sources of vitamin D3 (cholecalciferol), which is essential for calcium regulation and skeletal health. Deficiency can cause rickets, osteomalacia, and decreased bone mineral density, potentially leading to osteoporosis. Vitamin D3 may also offer protection against multiple sclerosis, diabetes, and hypertension [31,32,34]. The general recommendation is to consume at least 1000 IU of vitamin D per day, which corresponds to 25 µg [31]. Vitamin D3 (cholecalciferol) can also be photochemically produced in the skin from 7-dehydrocholesterol through exposure to sunlight [31,32]. Although fish intake is encouraged, the recommended amounts are generally insufficient to fully optimise vitamin D levels. For this reason, food fortification, such as adding vitamin D to margarine, is widely practised in numerous countries [32]. Vitamin E, a lipid-soluble antioxidant, protects cellular structures from oxidative damage, plays a role in anti-inflammatory processes, and may delay chronic diseases such as CVD and Alzheimer’s disease [34].

Energy metabolism requires water-soluble B-complex vitamins: thiamin (40–210 mg/100 g), niacin (3.6 mg/100 g), riboflavin, and cobalamin (1–9 mg/100 g) [15,16,51,52]. Niacin is essential for the metabolism of carbohydrates, lipids, and amino acids, and is further recognized for its immunomodulatory and antioxidant effects [53]. Riboflavin (vitamin B2) plays a key role in numerous metabolic processes and has demonstrated anti-inflammatory and antioxidant properties, particularly in the management of Crohn’s disease [52]. Vitamin B12 (cobalamin) is essential for proper DNA replication, erythropoiesis, and maintaining neurological function. Its deficiency has been associated with cognitive decline and CVD [34]. Fresh fish also contains limited vitamin C, which is nonetheless important for tissue repair, structural integrity, and enhancing iron absorption relevant to nervous system function [15].

Fish is a good source of minerals such as calcium, magnesium, phosphorus, iodine, selenium, iron, zinc, and copper [15,16,27]. Both species contain relatively little sodium but are notably high in potassium [16].

Iron from fish is highly bioavailable and is critical for hemoglobin synthesis and myoglobin in the blood, which are responsible for oxygen transport. Calcium supports bone and dental health, muscle contraction, nerve signalling, and blood coagulation [15,34]. Magnesium acts as a cofactor in over 300 enzymatic processes, including DNA and RNA synthesis, blood glucose control, and blood pressure regulation. It also contributes to calcium and potassium ion transport across membranes, supports muscle function, and may help reduce the risk of hypertension, CVD, and diabetes [34].

Zinc, a very important microelement, enhances wound healing, immune defence, and vision [15,34]. It is essential for taste and smell, and may protect against age-related macular degeneration. Copper acts as a cofactor in iron metabolism, red blood cell formation, immune response, neurodevelopment, and protection from oxidative damage. It may also play a protective role against CVD, Alzheimer’s disease, and dementia [34]. Iodine and selenium, abundant in seafood, are vital for thyroid hormone metabolism, cognitive development, and antioxidant defence [16,30,32,34]. Iodine is especially concentrated in lean fish and is necessary for hormonal metabolic activities [15,32,34]. Selenium also serves as a cofactor in antioxidant processes [32,34].

Since large fish are typically consumed for their fillets, removing viscera, bones, and organs reduces their overall micronutrient content compared to whole small fish. This was demonstrated by the authors of [54], who found that replacing small wild fish with larger farmed species decreased population-level intakes of essential micronutrients such as iron and calcium. Therefore, the consumption of whole small fish provides considerable amounts of various micronutrients, particularly minerals (I, Se, Fe, Zn, Ca, P, and K) and vitamins A, D, and B12, contributing significantly to dietary needs [27,55].

Other bioactive compounds in fish include choline, folic acid, chromium, phospholipids, and antioxidants [30,34]. Choline supports neurotransmitter synthesis, lipid metabolism, and brain development, and may reduce the risk of neurological and liver diseases. Folic acid supports DNA synthesis and amino acid metabolism, and may help prevent preterm birth, CVD, dementia, and congenital anomalies. Chromium enhances insulin action, regulates glucose and lipid metabolism, and may benefit diabetics [34].

### 3.4. Impact of Aquaculture on the Nutritional Profile of Sea Bass and Sea Bream

Aquaculture of sea bass and sea bream is of major economic importance in the Mediterranean region due to high consumer commercial interest [8,9,12,17,28]. In European aquaculture, gilthead sea bream is among the most significant marine finfish, with most of its production occurring in Mediterranean and Northern European regions, accounting for 4.1% of the main aquaculture species output [17]. Additionally, aquaculture is regarded as a sustainable and highly effective method for generating quality protein for human diets [15].

Fish feed is a critical determinant of fatty acid composition, particularly in aquaculture systems where feed composition is tightly controlled. Importantly, fish farming practices directly influence lipid profiles. In a study by Ofori-Mensah et al. [56], gilthead sea bream fed diets enriched with camelina seed oil or chia oil produced fillets containing 1373.4 mg and 1449.9 mg of EPA + DHA per 250 g serving, respectively, while maintaining favorable sensory attributes, including color and texture. These levels far exceed EFSA’s minimum daily recommendation [56].

In terms of nutritional quality, farmed sea bass has been found to be comparable to wild counterparts thereby helping to alleviate concerns about the nutritional adequacy of aquaculture products [57]. Although wild fish often show higher concentrations of EPA and DHA, farmed sea bass could also be a reliable source of these valuable *n*-3 fatty acids. Another *n*-3 fatty acid-DHA has been found in similarly high levels in both farmed and wild specimens [58]. A study by Di Marco et al. [59] reported that organic sea bream and sea bass had lower EPA and DHA values (sea bream: 0.93 vs. 1.25 g/100 g; sea bass: 0.73 vs. 1.28 g/100 g) compared to conventionally farmed counterparts.

## 4. Evidence-Based Health Benefits of Fish Consumption

Regular fish intake is recognized as beneficial in reducing risk and aiding the management of various chronic conditions, especially CVD, which remains the leading cause of death worldwide, accounting for over 40% of fatalities from non-communicable diseases [32,34,38,45]. However, it should be noted that the majority of this evidence is derived from studies on overall fish consumption and omega-3 fatty acids [60,61,62], while direct clinical evidence for specific aquaculture species remains limited. CVD includes a range of conditions, such as coronary heart disease (CHD) and stroke, with atherosclerosis as a key underlying mechanism [32,34]. Several lifestyle and metabolic factors, including tobacco use, poor diet, physical inactivity, excessive body weight, high blood pressure, and abnormal lipid profiles, significantly influence both the onset and progression of CVD [32,37,63]. Among these, hypertension stands out as one of the most prevalent global health concerns, responsible for an estimated 10.4 million deaths in 2017, with approximately 1.28 billion adults affected worldwide in 2023 [37,64]. A study [37] further demonstrated that dietary PUFAs can beneficially influence diastolic blood pressure independently of systolic pressure. Supplementation with AA and EPA, or dietary strategies that enhance the conversion of LA and ALA to AA and EPA, were associated with reduced diastolic pressure [37].

Many prospective cohort studies and meta-analyses on overall fish intake consistently confirm the cardio protective effects of regular fish consumption, particularly at intake levels of 2–4 servings per week [32,39,41]. Such consumption is associated with a 12% reduction in CVD mortality and a 21% reduction in CHD mortality [32,39,41,45,65,66]. Additionally, each 20 g/day increase in fish intake results in a 4% reduction in CVD mortality and a 7% reduction in CHD mortality [41,65,66]. Fish consumption is also associated with reduced risks of ischaemic stroke (12%), cerebrovascular events (6%), and myocardial infarction (4%) [34,41,67,68,69]. Moreover, eating fatty fish has a beneficial impact on lipid balance, as it contributes to lower triglyceride concentrations and increased HDL cholesterol levels [34,41,70].

The largest prospective cohort study to date, including over 420,000 participants from the National Institutes of Health AARP Diet and Health Study, demonstrated significant inverse associations between fish intake and CVD mortality [60]. Over a 16-year follow-up, both men and women exhibited 10% lower CVD mortality, with reductions reaching 15% in men and 18% in women across the extreme quintiles of EPA and DHA intake [36,60]. The GISSI-Prevenzione and JELIS trials, two of the largest randomized clinical trials, reported 10% and 19% reductions in major cardiovascular events, respectively, following fish oil supplementation [61,62]. Mozaffarian and Rimm [71] found that even modest fish consumption conferred a 36% reduction in coronary death and a 17% decrease in all-cause mortality, while the authors of [39] concluded that 250–566 mg/day may reduce CHD mortality by up to 37% [30,39,71].

Similarly, increased EPA and DHA levels were associated with up to 21% lower CHD risk in a five-year cohort study among Greenland Inuits [72]. Altogether, *n*-3 LC-PUFAs provide substantial cardiovascular protection, especially through their anti-inflammatory, anti-thrombotic, and lipid-modulating actions [32,33,36,41,42]. Findings by [39] suggest that eating fish and seafood two to three times per week, or at least one serving of fatty fish weekly, may help meet the daily requirements for *n*-3 LC-PUFAs.

Beyond cardiovascular outcomes, general fish consumption has been associated with a reduced risk of certain cancers and offers notable metabolic benefits, particularly through the intake of fatty fish [34,41,73]. Although lean fish contain lower overall fat levels, they still provide meaningful amounts of *n*-3 LC-PUFAs and may be beneficial in dietary strategies aimed at managing metabolic syndrome [32]. These effects may be further enhanced by bioactive compounds such as taurine, which has been shown to lower blood glucose and improve insulin sensitivity, thereby minimizing complications related to type 2 diabetes [31]. Additionally, regular fish intake has demonstrated protective effects against chronic inflammatory conditions such as Crohn’s disease, rheumatoid arthritis, inflammatory bowel disease and certain types of cancer [30,41,42,43]. Patients with such conditions often combine dietary and lifestyle changes with pharmacological treatment to manage gastrointestinal symptoms [43].

These associations are largely attributed to the anti-inflammatory properties of *n*-3 LC-PUFAs and other bioactive fish components [42]. An optimal *n*-6/*n*-3 ratio is essential for maintaining inflammation balance and preventing disease, and regular fish consumption helps improve this ratio in the diet [74,75]. While the majority of epidemiological and clinical evidence refers to general fish consumption, studies on specific aquaculture species, including seabream and seabass, indicate that they also possess favorable fatty acid profiles that may contribute to similar health benefits [74]. Pleadin et al. [74] analysed different fish species originated from aquaculture (sea bream, sea bass, dentex, and turbot) and found that all analysed species met the recommended *n*-3/*n*-6 ratios. By integrating into cellular membranes, *n*-3 LC-PUFAs support membrane integrity, regulate cytokine signalling, reduce oxidative stress, and promote metabolic homeostasis [32,41]. These effects occur through various biological pathways, including lipid metabolism, immune regulation, and neuroprotection [32,33,34].

Additionally, fish consumption has been associated with lower rates of depression, a reduced risk of cognitive disorders such as dementia and Alzheimer’s disease (with the greatest reductions of 21–29% observed at 2–4 servings per week), as well as other health benefits including enhanced immune function, improved infant neurodevelopment, better mental health, and metabolic regulation in adults [15,30,32,41,76,77]. Beyond these effects, epidemiological and preclinical studies indicate that DHA, a major component of fish oils, may be protective against dementia, Alzheimer’s disease, and macular degeneration [30]. In a study by [62], participants not taking antihypertensive medication or fish oil supplements showed a link between increased DHA in serum phospholipids and lower blood pressure in both clinical and ambulatory measurements. DHA is highly concentrated in brain and retinal tissue and has been demonstrated to be particularly important for brain and visual development, as well as neuronal activity [30].

Intake of fatty acids from the group of *n*-3 during pregnancy is strongly associated with a reduced risk of preterm birth and increased birth weight, while low seafood consumption during this critical period is linked to suboptimal neurodevelopmental outcomes in children, including impaired cognition, visual acuity, and fine motor skills [16,27,30,31,34,41]. The beneficial effects are mainly due to DHA, which is highly concentrated in the brain and retina and plays a vital role in the structural development of the nervous system in newborns, particularly during late gestation and the early postnatal period. Fatty acids from the group of *n*-3 LC-PUFAs are incorporated into cell membranes, where they influence membrane structure and function, and are considered essential nutrients for growth and neurodevelopment during pregnancy and early childhood [39]. Pregnant women, children, and vulnerable groups particularly benefit from regular fish intake, given the critical role of DHA in neurodevelopment and vision, with EFSA recommending 3–4 servings per week during pregnancy to support optimal fetal outcomes [39,50].

## 5. Nutrition and Health Claims on Fish from Aquaculture: Consumer Perception and Compliance Criteria

### 5.1. Information Valued by Consumers in Aquaculture Fish Labelling

Consumers in different markets express distinct preferences regarding the amount and type of information presented on fish product packaging. Altintzoglou and Nøstvold [78] found that Norwegian consumers perceived excessive information on the front of packaging as overwhelming and preferred a clearer view of the product itself. The authors suggested that only key information sought by consumers, such as shelf life, country of origin, and whether the product was previously frozen, should appear on the front, while additional mandatory or voluntary information could be placed on the back of the packaging or made available through supplementary sources such as leaflets or online platforms.

Preferences for labelling practices also vary by country. In the study by Krešić et al. [79] it was observed that Italian consumers favored packaging that included the method of production and eco-labels, whereas Croatian consumers placed greater emphasis on national quality labels. These findings underline the importance of tailoring packaging strategies and communication to specific consumer expectations in different markets.

### 5.2. Consumer Preferences for Fish from Aquaculture

Fish consumption is a complex behavior influenced by various cognitive and informational factors. Numerous studies have shown that consumer knowledge, both subjective and objective, plays a crucial role in explaining fish consumption patterns [79,80,81]. A higher level of knowledge about fish is consistently associated with more frequent consumption, greater interest in nutritional information, and more intensive use of informational cues when purchasing fish products [79,80]. Consumers with high levels of objective knowledge are more likely to interpret fish-related information correctly and exhibit less confusion or skepticism towards farmed fish [81]. Conversely, limited knowledge about aquaculture often leads to the perception that farmed fish are less favorable compared to their wild counterparts. This negative perception appears to result from poor communication about aquaculture, rather than from actual objective facts [81]. Therefore, enhancing consumer understanding through education and transparent labeling is crucial for promoting acceptance and growth of aquaculture, especially as Krešić et al. [82] reported that younger consumers, females, and those with higher income and education levels show a stronger preference for aquaculture products.

Cross-national studies have shown that consumer interest in nutritional information when purchasing fish is particularly strong in countries such as Greece, the United Kingdom, Romania, Portugal, and the Czech Republic [80]. Southern European countries, notably Greece, Portugal, and Italy, also reported the highest levels of fish consumption, which coincided with the highest levels of fish-related knowledge and interest in additional information, such as origin [80,83].

### 5.3. Consumer Trust in Nutrition and Health Claims

Consumer interest in nutrition and health claims on fish products is evident, with a willingness to pay a premium for products carrying such labels, particularly when they are associated with environmental or societal benefits, as in the case of sustainability labels. Despite this demonstrated interest, the actual level of consumer knowledge regarding the specific nutritional and health benefits of fish remains limited, suggesting a gap between perceived value and informed understanding [38].

Evidence also indicates that the credibility of structure–function-type claims (those linking a nutrient’s molecular characteristics to a specific metabolic function) depends largely on their consistency with consumers’ pre-existing mental representations of nutrient–function relationships. For instance, calcium is intuitively associated with bone health, while meat is linked to muscle development [84]. When such associations align with consumers’ expectations, claims tend to be perceived as more believable.

However, not everyone is equally open to nutrition and health claims. In a study involving French consumers, the majority expressed skepticism towards functional foods and associated claims, often citing lack of understanding, low visibility of claims on packaging, or even complete rejection based on the presence of such claims [84]. This highlights the importance of both message clarity and cultural context in shaping consumer response.

Additionally, prior beliefs and familiarity with certain nutrients or ingredients appear to play a more significant role in shaping perceptions of nutrition and health claims than the claims themselves. Consumers often use their existing knowledge to interpret health-related information, sometimes attributing additional meaning beyond what is explicitly stated. For example, a nutrient mentioned in a claim may be associated with disease prevention or general health improvement, regardless of the actual content of the claim [85]. This suggests that increasing public familiarity with key nutrients could improve both the comprehension and effectiveness of health-related messaging on fish products.

### 5.4. Nutrition and Health Claims: Definitions and Applications

According to European Regulation [22], a nutrition claim refers to a nutrient-related statement permitted only if listed in the Regulation’s Annex and meeting all specified conditions [86]. Claims include: content claims (e.g., “high fibre”), comparative claims (e.g., “reduced sugar”), and non-addition claims (e.g., “no added sugar”) [86].

A health claim, according to [22], is any claim that suggests the existence of a relationship between a food and health [23,86,87,88]. The Regulation includes three main types of health claims, which are further classified under Article 13 and Article 14. Article 13 covers general function claims (e.g., “calcium is needed for normal bone maintenance”), while Article 14 includes claims related to the reduction in disease risk and to children’s development and health [22,25]. Claims are permitted only if they are scientifically substantiated and meet the conditions outlined in the Regulation, including accompanying statements on the importance of a varied and balanced diet, consumption quantity, usage warnings, and relevant populations. Claims that suggest negative health consequences from not consuming a product or that include endorsements from health professionals are prohibited [22].

There is widespread consensus that claims must be clear, not misleading, and understandable to the average consumer, and must be scientifically supported [22,23,24]. EFSA has evaluated more than 900 submitted health claims and has published scientific opinions supporting approximately 125 of them [29]. However, many claims receive unfavorable opinions due to a lack of evidence for a cause-and-effect relationship [23,29]. The list of permitted health claims (other than Article 14 claims) is published in [89] and includes the nutrient or substance, the approved claim, usage conditions, and relevant EFSA documentation (EFSA Journal number and relevant list entry submitted to EFSA for assessment).

Despite comprehensive regulation, data on the actual application of claims on fish products are scarce. Studies show that simple claims such as “catch area” or “farmed in,” which are usually mandatory, are more easily recalled by consumers than voluntary claims, possibly due to their simplicity and familiarity. When too much information is presented on a label, recall and understanding decline—a phenomenon known as the “less is more” effect. Since health claims tend to be longer and more complex due to regulatory requirements, they may be less appealing to consumers and reduce purchase intent [90].

Citing earlier research, ref. [23] reported that 47.3% of food products in Ireland included nutrition claims and 17.8% featured health claims, figures notably higher than the 14% documented in Australia (2005), yet similar to the 49.7% observed in the United States (2000). Moreover, the formal definition of “healthy” was introduced more than three decades ago, when public health priorities focused on limiting total fat intake. Labelling a product as “healthy” is permitted only if it contains a significant amount of foods from the groups recommended in the 2015–2020 Dietary Guidelines for Americans and complies with limits on saturated fats, sodium, and added sugars [91].

### 5.5. Nutrient Composition of Gilthead Sea Bream and European Sea Bass for Authorized Nutrition and Health Claims

Accurate assessment of the nutritional composition of gilthead sea bream and European sea bass is essential for determining their eligibility to bear authorized nutrition and health claims under current regulatory frameworks. This section provides an overview of the available literature on nutrient levels in these species and discusses the extent to which they meet the thresholds required for authorized claims.

#### 5.5.1. Nutrition Claims: Criteria for Product Eligibility

To evaluate the potential of gilthead sea bream and European sea bass to meet the conditions for specific nutrition claims in accordance with EU regulations, a comparative overview of their nutrient composition was compiled from available literature sources. Table 1, Table 2, Table 3 and Table 4 present the reported ranges of macro- and micronutrient concentrations in both species, alongside the thresholds required for corresponding nutrition claims as defined by European Regulation [22]. For each nutrient, it is indicated whether the reported values meet the conditions necessary to bear the nutrition claim (stated as yes, no and potentially when claim is partially satisfied, i.e., when part of the range meets the conditions). References for the data ranges are listed on the furthest right column. The nutritional ranges presented in Table 1, Table 2, Table 3 and Table 4 are derived from multiple literature sources, including studies on aquaculture-raised, wild-caught, and commercially available (market) fish. In several cases, the origin of the fish was not explicitly specified, particularly in review articles. Therefore, the reported values represent aggregated ranges across different production systems rather than exclusively aquaculture-derived data.

**Table 1:** Micronutrient Levels in Gilthead Sea bream: Literature-Based Ranges, Claim Thresholds, and Eligibility for Nutrition Claims.

Mineral	Range	Criteria for *Source of*	Criteria for *High*	Meets Criteria for Nutrition Claim?	Reference
Calcium	19.2–94.3 mg/100 g	at least 120 mg/100 g	at least 240 mg/100 g	No	[9,11,17,74,92,93]
Magnesium	22.2–35.8 mg/100 g	at least 56.25 mg/100 g	at least 112.5 mg/100 g	No	[9,11,17,92,93]
Manganese	0.02–0.04 mg/100 g	at least 0.3 mg/100 g	at least 0.6 mg/100 g	No	[17,92,93]
Phosphorus	218–356 mg/100 g	at least 105 mg/100 g	at least 210 mg/100 g	Yes, both *Source of* and *High*	[9,11,17,74,92,93]
Selenium	23.6–40.0 µg/100 g	at least 8.25 µg/100 g	at least 16.5 µg/100 g	Yes, both *Source of* and *High*	[9,11,28,93,94]
Iron	0.35–1.01 mg/100 g	at least 2,1 mg/100 g	at least 4.2 mg/100 g	No	[11,17,92,93]
Zinc	0.11–0.72 mg/100 g	at least 1.5 mg/100 g	at least 3.0 mg/100 g	No	[9,11,17,92]
Copper	0.03–0.28 mg/100 g	at least 0.15 mg/100 g	at least 0.3 mg/100 g	Potentially–*Source of*	[11,17,92]
Potassium	270.0–424.5 mg/100 g	at least 300 mg/100 g	at least 600 mg/100 g	Potentially–*Source of*	[9,11,17,92,93]
		Criteria for *Low*	Criteria for *Very low*		
Sodium	28.9–100.0 mg/100 g	≤120 mg/100 g	≤40 mg/100 g	Yes–*Low*; potentially–*Very low*	[9,17,74,93]
Vitamin	Range	Criteria for *Source of*	Criteria for *High*	Meets Criteria for Nutrition Claim?	
Vitamin A	30–90 µg/100 g	at least 120 µg/100 g	at least 240 µg/100 g	No	[95,96]
Vitamin E	0.2–0.56 mg/100 g	at least 1.8 mg/100 g	at least 3.2 mg/100 g	No	
Vitamin B2	0.07–0.10 mg/100 g	at least 0.21 mg/100 g	at least 0.42 mg/100 g	No	[92,95]

**Table 2:** Macronutrient Levels in Gilthead Sea bream: Literature-Based Ranges, Claim Thresholds, and Eligibility for Nutrition Claims.

Nutrient	Range	Criteria for *Source of*	Criteria for *High*	Meets Criteria for Nutrition Claim?	Reference
Protein	16.0–22.5%	at least 12% of energy is provided by protein content	at least 20% of energy is provided by protein content	Yes, both *Source of* and *High*	[4,9,28,39,46,56,59,74,75,93,94,95,97,98,99,100,101]
ALA	0.05–0.33 g/100 g	at least 0.3 g/100 g/100 kcal	at least 0.6 g/100 g/100 kcal	Potentially–*Source of*	[4,28,99]
EPA + DHA	0.25–1.28 g/100 g	at least 0.04 g of EPA and DHA/100 g/100 kcal	at least 0.08 g of EPA and DHA/100 g/100 kcal	Yes, both *Source of* and *High*	[4,28,97,98,99]
		Criteria for Saturated fat-free	Criteria for Low saturated fat		
SFAs	0.57–1.99 g/100 g	≤0.1 g/100 g of combined SFAs and trans fatty acids	≤1.5 g/100 g of combined SFAs and trans fatty acids	Potentially–*Low saturated fat*	[4,28,98,99]
		Criteria for *High*			
MUFAs	32.01–54.66% of total fatty acids	at least 45% of all fatty acids are MUFAs + they provide more than 20% of energy		Potentially–*High*	[46,56,59,74,93,100,101]
PUFAs	24.10–40.97% of total fatty acids	at least 45% of all fatty acids are PUFAs + they provide more than 20% of energy		No	[9,47,63,95,96,101,102]
UFAs	70.42–78.76% of total fatty acids	at least 70% of all fatty acids are UFAs + they provide more than 20% of energy		Yes–*High*	

ALA—alpha-linolenic acid; EPA—eicosapentaenoic acid; DHA—docosahexaenoic acid; SFAs—saturated fatty acids; MUFAs—monounsaturated fatty acids; PUFAs—polyunsaturated fatty acids; UFAs—unsaturated fatty acids.

**Table 3:** Micronutrient Levels in European Sea bass: Literature-Based Ranges, Claim Thresholds, and Eligibility for Nutrition Claims.

Mineral	Range	Criteria for *Source of*	Criteria for *High*	Meets Criteria for Nutrition Claim?	Reference
Calcium	14.0–72.9 mg/100 g	at least 120 mg/100 g	at least 240 mg/100 g	No	[9,14,74,92]
Magnesium	30–34 mg/100 g	at least 56.25 mg/100 g	at least 112.5 mg/100 g	No	[9,14,93]
Manganese	0.02–0.06 mg/100 g	at least 0.3 mg/100 g	at least 0.6 mg/100 g	No	
Phosphorus	202.0–373.6 mg/100 g	at least 105 mg/100 g	at least 210 mg/100 g	Yes–*Source of*; potentially–*High*	[9,14,74,92]
Selenium	22–29 µg/100 g	at least 8.25 µg/100 g	at least 16.5 µg/100 g	Yes, both *Source of* and *High*	[9,92,102]
Iron	0.3–1.0 mg/100 g	at least 2.1 mg/100 g	at least 4.2 mg/100 g	No	[14,92,102]
Zinc	0.28–0.62 mg/100 g	at least 1.5 mg/100 g	at least 3.0 mg/100 g	No	[9,14,92]
Copper	0.04–0.18 mg/100 g	at least 0.15 mg/100 g	at least 0.3 mg/100 g	Potentially–*Source of*	[14,92,103]
Potassium	306.0–459.7 mg/100 g	at least 300 mg/100 g	at least 600 mg/100 g	Yes–*Source of*	[9,14,92]
		Criteria for *Low*	Criteria for *Very low*		
Sodium	70.6–139.0 mg/100 g	≤120 mg/100 g	≤40 mg/100 g	Potentially–*Low*	[9,14,74]
Vitamin		Criteria for *Source of*	Criteria for *High*	Meets Criteria for Nutrition Claim?	
Vitamin A	30–100 µg/100 g	at least 120 µg/100 g	at least 240 µg/100 g	No	[57,92,96]
Vitamin E	0.48–2.3 mg/100 g	at least 1.8 mg/100 g	at least 3.2 mg/100 g	Yes–*Source of*	[57,92,96,104]
Vitamin B2	0.15–0.20 mg/100 g	at least 0.21 mg/100 g	at least 0.42 mg/100 g	No	[57,92]

**Table 4:** Macronutrient Levels in European Sea bass: Literature-Based Ranges, Claim Thresholds, and Eligibility for Nutrition Claims.

Nutrient	Range	Criteria for *Source of*	Criteria for *High*	Meets Criteria for Nutrition Claim?	Reference
Protein	16.6–24.3%	at least 12% of energy is provided by protein content	at least 20% of energy is provided by protein content	Yes, both *Source of* and *High*	[5,9,14,33,57,58,59,74,92,104,105,106,107,108,109,110,111,112]
ALA	0.02–0.42 g/100 g	at least 0.3 g/100 g/100 kcal	at least 0.6 g/100 g/100 kcal	Potentially–*Source of*	[5,33,107,112]
EPA + DHA	0.47–1.37 g/100 g	at least 0.04 g of EPA and DHA/100 g/100 kcal	at least 0.08 g of EPA and DHA/100 g/100 kcal	Yes, both *Source of* and *High*	[5,33,107,111,112]
		Criteria for *Saturated fat-free*	Criteria for *Low saturated fat*		
SFAs	0.73–3.03 g/100 g	≤0.1 g/100 g of combined SFAs and trans fatty acids	≤1.5 g/100 g of combined SFAs and trans fatty acids	Potentially–*Low saturated fat*	[5,33,92,107,111,112]
		Criteria for *High*			
MUFAs	30.14–42.80% of total fatty acids	at least 45% of all fatty acids are MUFAs + they provide more than 20% of energy		No	[14,65,66,67,110,111,112]
PUFAs	32.00–39.60% of total fatty acids	at least 45% of all fatty acids are PUFAs + they provide more than 20% of energy		No	
UFAs	63.96–75.90% of total fatty acids	at least 70% of all fatty acids are UFAs + they provide more than 20% of energy		Potentially–*High*	

ALA—alpha-linolenic acid; EPA—eicosapentaenoic acid; DHA—docosahexaenoic acid; SFAs—saturated fatty acids; MUFAs—monounsaturated fatty acids; PUFAs—polyunsaturated fatty acids; UFAs—unsaturated fatty acids.

Based on the criteria established in Regulation [22] regarding nutrition claims made on foods, most vitamins and minerals present in gilthead sea bream do not reach the threshold required to bear a *Source of* nutrition claim (Table 1). Specifically, the concentrations of calcium, magnesium, manganese, iron, zinc, vitamin A, vitamin E, and vitamin B2 (riboflavin) are below the levels required for the claim *Source of [nutrient]*. However, phosphorus and selenium meet the criteria for both the standard claim *Source of* and the stricter claim *High*, indicating that sea bream is a reliable dietary source of these two essential minerals. In addition, copper and potassium show potential to meet the *Source of* claim, although their levels are insufficient to qualify for the *High* claim. Notably, the low sodium content of gilthead sea bream qualifies it for the claim *Low sodium/salt*, with the potential to reach the stricter claim *Very low sodium/salt* (Table 1).

The average protein content in gilthead sea bream is approximately 19.3 g per 100 g. When expressed as a percentage of total energy, based on an average energy content of 942 kJ per 100 g (calculated as the mean from the reported range in sea bream of 790–1094 kJ per 100 g [4,28,46,100]), protein contributes around 34.8% of total energy. This well exceeds the 20% threshold, qualifying sea bream for both the *Source of protein* and the stricter *High protein* nutrition claims. This confirms sea bream as a high-quality protein source with significant nutritional value. Additionally, the SFA content potentially meets the requirement for the *Low saturated fat* claim (Table 2).

The fatty acid profile of gilthead sea bream supports several potential nutrition claims. The sum of EPA and DHA concentrations clearly satisfies the criteria for the *High n-3 fatty acids* claim, which requires at least 0.08 g of EPA plus DHA per 100 g and per 100 kcal of product [22]. In contrast, ALA shows limited potential to fulfil the requirements for the *Source of n-3 fatty acids* claim, as its concentrations tend to be below the 0.3 g per 100 g and 100 kcal threshold. Regarding other lipid fractions, MUFAs display potential to qualify for the *High monounsaturated fat* claim, while PUFAs do not meet the required level for the corresponding *High* claim. Importantly, total UFAs in sea bream are sufficient to support the *High unsaturated fat* nutrition claim, which may be particularly relevant for consumer communication strategies focused on heart health (Table 2).

The micronutrient profile of European sea bass shows that the concentrations of calcium, magnesium, manganese, iron, zinc, vitamin A, and vitamin B2 (riboflavin) are below the minimum levels required to carry the nutrition claim *Source of [nutrient]* (Table 3). However, sea bass is a reliable dietary source selenium, meeting the thresholds for both the standard claim *Source of* and the stricter claim *High*. Additionally, copper shows potential to meet the *Source of cooper* claim, although not the stricter *High copper* claim. Phosphorus and potassium also meet the requirement for the *Source of* claim, but not the *High* version. Notably, sea bass has a low sodium content, which gives it potential to meet the *Low sodium/salt* claim, though not the more stringent *Very low sodium/salt* claim. Among the vitamins, only vitamin E shows potential to qualify for the *Source of vitamin E* nutrition claim.

European sea bass is a protein-rich food, with an average protein content of 20.5 g per 100 g. When calculated as a percentage of energy (based on an average energy value of 876.5 kJ per 100 g, determined as the mean from the reported range for energy in sea bass, 703–1050 kJ/100 g [5,33,92,108,109]), proteins contribute approximately 39.8% of the total energy. This significantly exceeds the thresholds for both the *Source of protein* claim (≥12% of energy from protein) and the *High protein* claim (≥20%), thus qualifying sea bass for both claims. However, the content of SFAs has the potential to meet the criteria for the claim *Low saturated fat* (Table 4).

European sea bass presents a favorable fatty acid profile in terms of *n*-3 content. The levels of EPA and DHA satisfy the stricter nutrition claim *High in n-3 fatty acids*, as their combined content exceeds the minimum threshold of 0.08 g per 100 g and 100 kcal [22]. In contrast, ALA shows limited potential to meet the threshold for the *Source of n-3 fatty acids* claim. Regarding other fatty acid groups, MUFAs and PUFAs do not fulfil the criteria for the claim *High* in their respective categories, as they fall short of the required 45% of total fatty acids and must also contribute over 20% of total energy. However, the overall content of UFAs shows potential to meet the criteria for the *High in unsaturated fat* claim, which requires UFAs to represent at least 70% of total fatty acids and to contribute more than 20% of energy (Table 4).

#### 5.5.2. Health Claims: Criteria for Product Eligibility

To carry a health claim from the list of permitted health claims made on food [89] food must first satisfy the associated nutrition claim [22].

In the case of ALA, the claim “ALA contributes to the maintenance of normal blood cholesterol levels” can only be made if the nutrition claim *Source of n-3 fatty acids* is achieved. While certain sea bream and sea bass samples met the minimum threshold for ALA (≥0.3 g/100 g), this was not consistent across the values reported in the literature. Given the seasonal variability in ALA content, this health claim should be applied cautiously and only when batch-specific analysis confirms sufficient levels.

Neither sea bream nor sea bass met the minimum threshold to bear the nutrition claim *Source of calcium* and thus cannot carry any calcium-related health claims (Table 1 and Table 3).

For phosphorus, all available data for both sea bream and sea bass satisfy the criteria for the claim *Source of phosphorus* and even *High phosphorus* (Table 1 and Table 3). Consequently, they are eligible to bear the health claim: “Phosphorus contributes to normal energy-yielding metabolism, the function of cell membranes, and the maintenance of normal bones and teeth” (Table 2 and Table 4).

Both sea bream and sea bass exhibit DHA concentrations exceeding 0.04 g/100 g, meeting the criteria for the authorized health claim: “DHA contributes to the maintenance of normal brain function and normal vision”. Likewise, their combined EPA and DHA content supports the claim: “EPA and DHA contribute to the normal function of the heart”. This is supported by the fact that all literature data achieved the stricter nutrition claim *High n-3 fatty acids*.

Additional health claims related to EPA and DHA concentrations include: “Daily intake of 2 g of EPA and DHA combined contributes to the maintenance of normal blood pressure” and “Daily intake of 3 g of EPA and DHA combined contributes to the normal function of the heart”. However, based on the upper range of EPA + DHA content in these fish species, achieving the recommended weekly intake would require approximately eight portions (assuming an average fillet portion size of 125 g), which is impractical. Consequently, the health claim for DHA regarding the maintenance of normal blood triglyceride levels cannot be supported by either sea bream or sea bass.

In some reported values, particularly those for sea bass, the sodium content was low enough to satisfy the nutrition claim *Low sodium/salt* (Table 3) which qualifies those products for the health claim: “Reducing sodium consumption contributes to the maintenance of normal blood pressure”. However, as this was not consistent across reported values, product-specific verification is recommended.

Sea bream demonstrated a greater capacity than sea bass to satisfy the conditions for the health claim related to low or reduced saturated fat: “Reducing consumption of saturated fat contributes to the maintenance of normal blood cholesterol levels”, as most reported values met the nutrition claim for low saturated fat.

Literature data for UFAs in sea bream consistently met the criteria for the nutrition claim *High unsaturated fat* (Table 2), thus allowing it to bear the health claim: “Replacing saturated fat with unsaturated fat contributes to the maintenance of normal blood cholesterol levels”.

Sea bass shows potential to meet this requirement, although seasonal variability should be considered, and the health claim should only be used when supported by analytical data.

Both seabream and sea bass are rich in high-quality protein, with more than 20% of total energy derived from protein, enabling the application of the health claim: “Protein contributes to the maintenance of muscle mass and normal bones”.

## 6. Conclusions

Fish from aquaculture, particularly gilthead sea bream and European sea bass, play a crucial role in providing nutritionally valuable and safe fish to the market. Comprehensive analysis of literature data confirms that both species offer high-quality protein and favorable fatty acid profiles, with EPA + DHA levels consistently meeting criteria for *High n-3 fatty acids*. Both species are rich in phosphorus and selenium, qualifying for *High* claims, while copper and potassium meet *Source of* thresholds. Sea bream additionally meets *Very low sodium/salt* and *Low saturated fat* claims, whereas sea bass generally qualifies for *Low sodium/salt*.

These attributes support several authorized health claims, particularly those related to cardiovascular, cognitive, visual, and bone health. Claims for nutrients such as calcium or vitamin A are not applicable, and some, including those for sodium or ALA, require cautious use due to seasonal and production variability.

In addition to their nutritional value, consumer perception remains a key factor influencing the acceptance of aquaculture fish. While nutrition and health claims can help communicate the benefits of sea bream and sea bass, they may not fully address concerns related to farmed fish. In this context, sustainability certifications, such as those provided by Aquaculture Stewardship Council (ASC), Best Aquaculture Practices (BAP), or Global G.A.P. (GGN), may further strengthen consumer trust. Combining clear nutritional communication with transparent sustainability practices could therefore support greater consumer acceptance and contribute to more sustainable food systems.

In summary, although some nutritional differences exist between farmed and wild sea bream and sea bass, both provide substantial amounts of high-quality protein and beneficial lipids, supporting their relevance in a health-promoting diet. Clear communication of these benefits, alongside highlighting the nutritional equivalence of farmed and wild fish, may reduce consumer skepticism and encourage greater inclusion of aquaculture fish in balanced dietary patterns.

## Data Availability

No new data were created or analysed in this study. Data sharing is not applicable to this article.

## Abbreviations

The following abbreviations are used in this manuscript:

LC-PUFA	Long-chain polyunsaturated fatty acid
EPA	Eicosapentaenoic acid
DHA	Docosahexaenoic acid
PUFA	Polyunsaturated fatty acid
*n*-3	Omega-3
SFA	Saturated fatty acid
MUFA	Monounsaturated fatty acid
*n*-6	Omega-6
CVD	Cardiovascular disease
ALA	Alpha-linolenic acid
DPA	Docosapentaenoic acid
LA	Linoleic acid
AA	Arachidonic acid
EFSA	European Food Safety Authority
CHD	Coronary heart disease
UFA	Unsaturated fatty acid

## References

[1] Tocher D.R., Betancor M.B., Sprague M., Olsen R.E., Napier J.A. (2019). Omega-3 Long-Chain Polyunsaturated Fatty Acids, EPA and DHA: Bridging the Gap between Supply and Demand. Nutrients.

[2] FAO (2020). The State of World Fisheries and Aquaculture 2020; Sustainability in Action.

[3] Alfonso S., Mente E., Fiocchi E., Manfrin A., Dimitroglou A., Papaharisis L., Barkas D., Toomey L., Boscarato M., Losasso C. (2023). Growth Performance, Gut Microbiota Composition, Health and Welfare of European Sea Bass (*Dicentrarchus labrax*) Fed an Environmentally and Economically Sustainable Low Marine Protein Diet in Sea Cages. Sci. Rep..

[4] Filipa-Silva A., Marques A., Salgado M.A., Abreu H., Dias J., Valente L.M.P. (2023). Exploring Alternative Marine Lipid Sources as Substitutes for Fish Oil in Farmed Sea Bass (*Dicentrarchus labrax*) and Their Influence on Organoleptic, Chemical, and Nutritional Properties during Cold Storage. Front. Sustain. Food Syst..

[5] Mota C.S.C., Pinto O., Sá T., Ferreira M., Delerue-Matos C., Cabrita A.R.J., Almeida A., Abreu H., Silva J., Fonseca A.J.M. (2023). A Commercial Blend of Macroalgae and Microalgae Promotes Digestibility, Growth Performance, and Muscle Nutritional Value of European Seabass (*Dicentrarchus labrax* L.) Juveniles. Front. Nutr..

[6] Xu H., Turchini G.M., Francis D.S., Liang M., Mock T.S., Rombenso A., Ai Q. (2020). Are Fish What They Eat? A Fatty Acid’s Perspective. Prog. Lipid Res..

[7] Santigosa E., Brambilla F., Milanese L. (2021). Microalgae Oil as an Effective Alternative Source of EPA and DHA for Gilthead Seabream (*Sparus aurata*) Aquaculture. Animals.

[8] FAO (2024). The State of World Fisheries and Aquaculture 2024—Blue Transformation in Action.

[9] Erkan N., Ozden O. (2007). Proximate Composition and Mineral Contents in Aqua Cultured Sea Bass (*Dicentrarchus labrax*), Sea Bream (*Sparus aurata*) Analyzed by ICP-MS. Food Chem..

[10] Enes P., Panserat S., Kaushik S., Oliva-Teles A. (2011). Dietary Carbohydrate Utilization by European Sea Bass (*Dicentrarchus labrax* L.) and Gilthead Sea Bream (*Sparus aurata* L.) Juveniles. Rev. Fish. Sci..

[11] Öztürk D.K., Baki B., Karayücel İ., Öztürk R., Gören G.U., Karayücel S. (2019). Determination of Seasonal Vitamin and Mineral Contents of Sea Bream (*Sparus aurata* L., 1758) Cultured in Net Cages in Central Black Sea Region. Biol. Trace Elem. Res..

[12] Rocha C.P., Cabral H.N., Nunes C., Coimbra M.A., Gonçalves F.J.M., Marques J.C., Gonçalves A.M.M. (2019). Biochemical Impacts in Adult and Juvenile Farmed European Seabass and Gilthead Seabream from Semi-Intensive Aquaculture of Southern European Estuarine Systems. Environ. Sci. Pollut. Res..

[13] Muniesa A., Basurco B., Aguilera C., Furones D., Reverté C., Sanjuan-Vilaplana A., Jansen M.D., Brun E., Tavornpanich S. (2020). Mapping the Knowledge of the Main Diseases Affecting Sea Bass and Sea Bream in Mediterranean. Transbound. Emerg. Dis..

[14] Munekata P.E.S., Pateiro M., Domínguez R., Zhou J., Barba F.J., Lorenzo J.M. (2020). Nutritional Characterization of Sea Bass Processing By-Products. Biomolecules.

[15] Maulu S., Nawanzi K., Abdel-Tawwab M., Khalil H.S. (2021). Fish Nutritional Value as an Approach to Children’s Nutrition. Front. Nutr..

[16] Gil A., Gil F. (2015). Fish, a Mediterranean Source of n -3 PUFA: Benefits Do Not Justify Limiting Consumption. Br. J. Nutr..

[17] Pateiro M., Munekata P.E.S., Domínguez R., Wang M., Barba F.J., Bermúdez R., Lorenzo J.M. (2020). Nutritional Profiling and the Value of Processing By-Products from Gilthead Sea Bream (*Sparus aurata*). Mar. Drugs.

[18] Banovic M., Reinders M.J., Claret A., Guerrero L., Krystallis A. (2019). A Cross-Cultural Perspective on Impact of Health and Nutrition Claims, Country-of-Origin and Eco-Label on Consumer Choice of New Aquaculture Products. Food Res. Int..

[19] Krešić G., Dujmić E., Lončarić D., Zrnčić S., Liović N., Pleadin J. (2022). Profiling of Croatian Consumers Based on Their Intention to Consume Farmed Fish. Foods.

[20] Krešić G., Dujmić E., Lončarić D., Zrnčić S., Liović N., Pleadin J. (2023). Determinants of White and Fatty Fish Consumption by Croatian and Italian Consumers. Br. Food J..

[21] Cantillo J., Martín J.C., Román C. (2020). Discrete Choice Experiments in the Analysis of Consumers’ Preferences for Finfish Products: A Systematic Literature Review. Food Qual. Prefer..

[22] Regulation (EC) No 1924/2006 of The European Parliament and of The Council of 20 December 2006 on Nutrition and Health Claims Made on Foods. https://eur-lex.europa.eu/eli/reg/2006/1924/2014-12-13/eng.

[23] Lalor F., Kennedy J., Flynn M.A., Wall P.G. (2010). A Study of Nutrition and Health Claims—A Snapshot of What’s on the Irish Market. Public Health Nutr..

[24] Verhagen H., Vos E., Francl S., Heinonen M., Van Loveren H. (2010). Status of Nutrition and Health Claims in Europe. Arch. Biochem. Biophys..

[25] Laser Reuterswärd A. (2007). The New EC Regulation on Nutrition and Health Claims on Foods. Scand. J. Food Nutr..

[26] Kumar K. (2025). Aquatic Foods in Human Health, Nutrition, and Overall Welfare. Clin. Nutr. Open Sci..

[27] Thilsted S.H., Thorne-Lyman A., Webb P., Bogard J.R., Subasinghe R., Phillips M.J., Allison E.H. (2016). Sustaining Healthy Diets: The Role of Capture Fisheries and Aquaculture for Improving Nutrition in the Post-2015 Era. Food Policy.

[28] Ferreira M., Ribeiro P.C., Ribeiro L., Barata M., Domingues V.F., Sousa S., Soares C., Marques A., Pousão-Ferreira P., Dias J. (2022). Biofortified Diets Containing Algae and Selenised Yeast: Effects on Growth Performance, Nutrient Utilization, and Tissue Composition of Gilthead Seabream (*Sparus aurata*). Front. Physiol..

[29] EFSA Science, Safe Food, Sustainability. https://www.efsa.europa.eu/en.

[30] Larsen R., Eilertsen K.-E., Elvevoll E.O. (2011). Health Benefits of Marine Foods and Ingredients. Biotechnol. Adv..

[31] Tilami S.K., Sampels S. (2018). Nutritional Value of Fish: Lipids, Proteins, Vitamins, and Minerals. Rev. Fish. Sci. Aquac..

[32] Tørris C., Småstuen M.C., Molin M. (2018). Nutrients in Fish and Possible Associations with Cardiovascular Disease Risk Factors in Metabolic Syndrome. Nutrients.

[33] Marques A., Canada P., Costa C., Basto A., Piloto F., Salgado M.A., Abreu H., Dias J., Valente L.M.P. (2023). Replacement of Fish Oil by Alternative N-3 LC-PUFA Rich Lipid Sources in Diets for European Sea Bass (*Dicentrarchus labrax*). Front. Mar. Sci..

[34] Tacon A.G.J., Lemos D., Metian M. (2020). Fish for Health: Improved Nutritional Quality of Cultured Fish for Human Consumption. Rev. Fish. Sci. Aquac..

[35] FAO, WHO (2024). Report of the Joint FAO/WHO Expert Consultation on the Risks and Benefits of Fish Consumption.

[36] Innes J.K., Calder P.C. (2020). Marine Omega-3 (N-3) Fatty Acids for Cardiovascular Health: An Update for 2020. Int. J. Mol. Sci..

[37] Ma M., Yang F., Wang Z., Bao Q., Shen J., Xie X. (2021). Association of Plasma Polyunsaturated Fatty Acids with Arterial Blood Pressure: A Mendelian Randomization Study. Medicine.

[38] Menozzi D., Nguyen T.T., Sogari G., Taskov D., Lucas S., Castro-Rial J.L.S., Mora C. (2020). Consumers’ Preferences and Willingness to Pay for Fish Products with Health and Environmental Labels: Evidence from Five European Countries. Nutrients.

[39] Mesa M., Gil F., Olmedo P., Gil A. (2021). Nutritional Importance of Selected Fresh Fishes, Shrimps and Mollusks to Meet Compliance with Nutritional Guidelines of n-3 LC-PUFA Intake in Spain. Nutrients.

[40] Basto A., Marques A., Silva A., Sá T., Sousa V., Oliveira M.B.P.P., Aires T., Valente L.M.P. (2023). Nutritional, Organoleptic and Sensory Quality of Market-Sized European Sea Bass (*Dicentrarchus labrax*) Fed Defatted *Tenebrio Molitor* Larvae Meal as Main Protein Source. Aquaculture.

[41] Li N., Wu X., Zhuang W., Xia L., Chen Y., Wu C., Rao Z., Du L., Zhao R., Yi M. (2020). Fish Consumption and Multiple Health Outcomes: Umbrella Review. Trends Food Sci..

[42] Lenihan-Geels G., Bishop K., Ferguson L. (2013). Alternative Sources of Omega-3 Fats: Can We Find a Sustainable Substitute for Fish?. Nutrients.

[43] Gura K.M., Chan A., Zong W., Pai N., Duro D. (2024). From the Kitchen to the Medicine Cabinet: Examples of Food Products and Supplements Used for Therapeutic Intent. J. Pediatr. Gastroenterol. Nutr..

[44] Metherel A.H., Irfan M., Klingel S.L., Mutch D.M., Bazinet R.P. (2021). Higher Increase in Plasma DHA in Females Compared to Males Following EPA Supplementation May Be Influenced by a Polymorphism in ELOVL2: An Exploratory Study. Lipids.

[45] AbuMweis S., Jew S., Tayyem R., Agraib L. (2018). Eicosapentaenoic Acid and Docosahexaenoic Acid Containing Supplements Modulate Risk Factors for Cardiovascular Disease: A Meta-analysis of Randomised Placebo-control Human Clinical Trials. J. Human Nutr. Diet..

[46] Torno C., Staats S., Fickler A., De Pascual-Teresa S., Soledad Izquierdo M., Rimbach G., Schulz C. (2019). Combined Effects of Nutritional, Biochemical and Environmental Stimuli on Growth Performance and Fatty Acid Composition of Gilthead Sea Bream (*Sparus aurata*). PLoS ONE.

[47] Skalli A., Robin J.H., Le Bayon N., Le Delliou H., Person-Le Ruyet J. (2006). Impact of Essential Fatty Acid Deficiency and Temperature on Tissues’ Fatty Acid Composition of European Sea Bass (*Dicentrarchus labrax*). Aquaculture.

[48] GOED EPA & DHA Intake Recommendations. GOED—Global Organisation for EP and DHA Omega-3s. https://goedomega3.com/intake-recommendations.

[49] Zhou Q., Xu L., Xu Y., Xue Q., Xue C., Jiang X., Wen Y. (2025). Systematically Investigating the Qualities of Commercial Encapsulated and Industrial-Grade Bulk Fish Oils in the Chinese Market. Foods.

[50] EFSA Panel on Dietetic Products, Nutrition and Allergies (NDA) (2012). Scientific Opinion on the Tolerable Upper Intake Level of Eicosapentaenoic Acid (EPA), Docosahexaenoic Acid (DHA) and Docosapentaenoic Acid (DPA). EFSA J..

[51] Ceylan Z., Yaman M., Sağdıç O., Karabulut E., Yilmaz M.T. (2018). Effect of Electrospun Thymol-Loaded Nanofiber Coating on Vitamin B Profile of Gilthead Sea Bream Fillets (*Sparus aurata*). LWT.

[52] von Martels J.Z.H., Bourgonje A.R., Klaassen M.A.Y., Alkhalifah H.A.A., Sadaghian Sadabad M., Vich Vila A., Gacesa R., Gabriëls R.Y., Steinert R.E., Jansen B.H. (2020). Riboflavin Supplementation in Patients with Crohn’s Disease [the RISE-UP Study]. J. Crohn’s Colitis.

[53] Ge X., Pan J., Yongyut P., Jintasataporn O., Deng J., Mai K., Zhang Y. (2024). Effect of Dietary Niacin on Immune Response, Apoptosis and Microbial Community in the Intestine of Juvenile Turbot (*Scophthalmus maximus* L.). Aquac. Rep..

[54] Bogard J.R., Farook S., Marks G.C., Waid J., Belton B., Ali M., Toufique K., Mamun A., Thilsted S.H. (2017). Higher Fish but Lower Micronutrient Intakes: Temporal Changes in Fish Consumption from Capture Fisheries and Aquaculture in Bangladesh. PLoS ONE.

[55] Hasselberg A.E., Aakre I., Scholtens J., Overå R., Kolding J., Bank M.S., Atter A., Kjellevold M. (2020). Fish for Food and Nutrition Security in Ghana: Challenges and Opportunities. Glob. Food Secur..

[56] Ofori-Mensah S., Yıldız M., Arslan M., Ünal Şengör G.F., Kahraman T., Gelibolu S., Kaplan Ç. (2022). Replacement of Fish Oil by ALA-Rich Vegetable Oils in Diets of Gilthead Sea Bream: Effect on Final Eating Quality. Eur. J. Lipid Sci. Technol..

[57] Baki B., Gonener S., Kaya D. (2015). Comparison of Food, Amino Acid and Fatty Acid Compositions of Wild and Cultivated Sea Bass (*Dicentrarchus labrax* L., 1758). Turk. J. Fish. Aquat. Sci..

[58] Tarricone S., Caputi Jambrenghi A., Cagnetta P., Ragni M. (2022). Wild and Farmed Sea Bass (*Dicentrarchus labrax*): Comparison of Biometry Traits, Chemical and Fatty Acid Composition of Fillets. Fishes.

[59] Di Marco P., Petochi T., Marino G., Priori A., Finoia M.G., Tomassetti P., Porrello S., Giorgi G., Lupi P., Bonelli A. (2017). Insights into Organic Farming of European Sea Bass *Dicentrarchus labrax* and Gilthead Sea Bream *Sparus aurata* through the Assessment of Environmental Impact, Growth Performance, Fish Welfare and Product Quality. Aquaculture.

[60] Zhang Y., Zhuang P., He W., Chen J.N., Wang W.Q., Freedman N.D., Abnet C.C., Wang J.B., Jiao J.J. (2018). Association of Fish and Long-chain Omega-3 Fatty Acids Intakes with Total and Cause-specific Mortality: Prospective Analysis of 421 309 Individuals. J. Intern. Med..

[61] Mozaffarian D. (2007). JELIS, Fish Oil, and Cardiac Events. Lancet.

[62] Liu J.C., Conklin S.M., Manuck S.B., Yao J.K., Muldoon M.F. (2011). Long-Chain Omega-3 Fatty Acids and Blood Pressure. Am. J. Hypertens..

[63] Raatz S.K., Silverstein J.T., Jahns L., Picklo M.J. (2013). Issues of Fish Consumption for Cardiovascular Disease Risk Reduction. Nutrients.

[64] WHO Hypertension. WHO—World Health Organization. https://www.who.int/news-room/fact-sheets/detail/hypertension.

[65] He K., Song Y., Daviglus M.L., Liu K., Van Horn L., Dyer A.R., Greenland P. (2004). Accumulated Evidence on Fish Consumption and Coronary Heart Disease Mortality: A Meta-Analysis of Cohort Studies. Circulation.

[66] Jayedi A., Shab-Bidar S., Eimeri S., Djafarian K. (2018). Fish Consumption and Risk of All-Cause and Cardiovascular Mortality: A Dose–Response Meta-Analysis of Prospective Observational Studies. Public Health Nutr..

[67] Chowdhury R., Stevens S., Gorman D., Pan A., Warnakula S., Chowdhury S., Ward H., Johnson L., Crowe F., Hu F.B. (2012). Association between Fish Consumption, Long Chain Omega 3 Fatty Acids, and Risk of Cerebrovascular Disease: Systematic Review and Meta-Analysis. BMJ.

[68] Jayedi A., Zargar M.S., Shab-Bidar S. (2019). Fish Consumption and Risk of Myocardial Infarction: A Systematic Review and Dose-Response Meta-Analysis Suggests a Regional Difference. Nutr. Res..

[69] Zhao W., Tang H., Yang X., Luo X., Wang X., Shao C., He J. (2019). Fish Consumption and Stroke Risk: A Meta-Analysis of Prospective Cohort Studies. J. Stroke Cerebrovasc. Dis..

[70] Alhassan A., Young J., Lean M.E.J., Lara J. (2017). Consumption of Fish and Vascular Risk Factors: A Systematic Review and Meta-Analysis of Intervention Studies. Atherosclerosis.

[71] Mozaffarian D., Rimm E.B. (2006). Fish Intake, Contaminants, and Human Health: Evaluating the Risks and the Benefits. JAMA.

[72] Senftleber N.K., Albrechtsen A., Lauritzen L., Larsen C.L., Bjerregaard P., Diaz L.J., Rønn P.F., Jørgensen M.E. (2020). Omega-3 Fatty Acids and Risk of Cardiovascular Disease in Inuit: First Prospective Cohort Study. Atherosclerosis.

[73] Zhang M., Picard-Deland E., Marette A. (2013). Fish and Marine Omega-3 Polyunsatured Fatty Acid Consumption and Incidence of Type 2 Diabetes: A Systematic Review and Meta-Analysis. Int. J. Endocrinol..

[74] Pleadin J., Lešić T., Krešić G., Barić R., Bogdanović T., Oraić D., Vulić A., Legac A., Zrnčić S. (2017). Nutritional Quality of Different Fish Species Farmed in the Adriatic Sea. Ital. J. Food Sci.

[75] Ünal-Şengör G.F., Yildiz M., Metin Ö., Ofori-Mensah S., Ceylan Z. (2025). Compositions of Gilthead Sea Bream (*Sparus aurata* Linnaeus, 1758) from Different Culture Systems. Aquac. Int..

[76] Ruxton C.H.S. (2011). The Benefits of Fish Consumption. Nutr. Bull..

[77] Li F., Liu X., Zhang D. (2016). Fish Consumption and Risk of Depression: A Meta-Analysis. J. Epidemiol. Community Health.

[78] Altintzoglou T., Nøstvold B.H. (2014). Labelling Fish Products to Fulfil Norwegian Consumers’ Needs for Information. Br. Food J..

[79] Krešić G., Dujmić E., Lončarić D., Zrnčić S., Liović N., Pleadin J. (2022). Fish Consumption: Influence of Knowledge, Product Information, and Satisfaction with Product Attributes. Nutrients.

[80] Pieniak Z., Vanhonacker F., Verbeke W. (2013). Consumer Knowledge and Use of Information about Fish and Aquaculture. Food Policy.

[81] Hoque M.Z., Alam M.N. (2020). Consumers’ Knowledge Discrepancy and Confusion in Intent to Purchase Farmed Fish. Br. Food J..

[82] Krešić G., Dujmić E., Lončarić D., Buneta A., Liović N., Zrnčić S., Pleadin J. (2020). Factors Affecting Consumers’ Preferences for Products from Aquaculture. Croat. J. Food Sci. Technol..

[83] Menozzi D., Wongprawmas R., Sogari G., Gai F., Parisi G., Mora C. (2023). The Role of Objective and Subjective Knowledge on the Attitude and Intention of Italian Consumers to Purchase Farmed and Wild Fish. Agric. Food Econ..

[84] Masson E., Debucquet G., Fischler C., Merdji M. (2016). French Consumers’ Perceptions of Nutrition and Health Claims: A Psychosocial-Anthropological Approach. Appetite.

[85] Hodgkins C.E., Egan B., Peacock M., Klepacz N., Miklavec K., Pravst I., Pohar J., Gracia A., Groeppel-Klein A., Rayner M. (2019). Understanding How Consumers Categorise Health Related Claims on Foods: A Consumer-Derived Typology of Health-Related Claims. Nutrients.

[86] Lazíková J., Rumanovská L. (2022). Nutrition and Health Claims on Foods in the EU Legislation. Jurid. Trib..

[87] EFSA Panel on Dietetic Products, Nutrition, and Allergies (NDA) (2010). Scientific Opinion on the Substantiation of Health Claims Related to Docosahexaenoic Acid (DHA) and Maintenance of Normal (Fasting) Blood Concentrations of Triglycerides (ID 533, 691, 3150), Protection of Blood Lipids from Oxidative Damage (ID 630), Contribution to the Maintenance or Achievement of a Normal Body Weight (ID 629), Brain, Eye and Nerve Development (ID 627, 689, 704, 742, 3148, 3151), Maintenance of Normal Brain Function (ID 565, 626, 631, 689, 690, 704, 742, 3148, 3151), Maintenance of Normal Vision (ID 627, 632, 743, 3149) and Maintenance of Normal Spermatozoa Motility (ID 628) Pursuant to Article 13(1) of Regulation (EC) No 1924/2006. EFSA J..

[88] EFSA Panel on Dietetic Products, Nutrition, and Allergies (NDA) (2010). Scientific Opinion on Dietary Reference Values for Fats, Including Saturated Fatty Acids, Polyunsaturated Fatty Acids, Monounsaturated Fatty Acids, Trans Fatty Acids, and Cholesterol. EFSA J..

[89] Commission Regulation (EU) Consolidated TEXT: 32012R0432—EN—19.08.2024. https://eur-lex.europa.eu/eli/reg/2012/432/2024-08-19/eng.

[90] Bogliacino F., Charris R., Codagnone C., Folkvord F., Gaskell G., Gómez C., Liva G., Montealegre F. (2023). Less Is More: Information Overload in the Labelling of Fish and Aquaculture Products. Food Policy.

[91] Belarmino E.H., Carfagno M., Kam L., Ifeagwu K.-C., Nelson M.E., Seguin-Fowler R.A. (2024). Consideration of Nutrition and Sustainability in Public Definitions of ‘Healthy’ Food: An Analysis of Submissions to the US FDA. Public Health Nutr..

[92] Seafish. Seafood for Health. https://www.seafish.org/seafood-for-life/seafood-for-health/.

[93] Oteri M., Chiofalo B., Maricchiolo G., Toscano G., Nalbone L., Lo Presti V., Di Rosa A.R. (2022). Black Soldier Fly Larvae Meal in the Diet of Gilthead Sea Bream: Effect on Chemical and Microbiological Quality of Filets. Front. Nutr..

[94] Mechlaoui M., Dominguez D., Robaina L., Geraert P.-A., Kaushik S., Saleh R., Briens M., Montero D., Izquierdo M. (2019). Effects of Different Dietary Selenium Sources on Growth Performance, Liver and Muscle Composition, Antioxidant Status, Stress Response and Expression of Related Genes in Gilthead Seabream (*Sparus aurata*). Aquaculture.

[95] Kaba N., Yucel S., Baki B. (2009). Comparative Analysis of Nutritive Composition, Fatty Acids, Amino Acids and Vitamin Contents of Wild and Cultured Gilthead Seabream (*Sparus aurata* L. 1758). J. Anim. Vet. Adv..

[96] Harlioğlu A.G., Yilmaz Ö., Oray I.K., Aydin S. (2016). A Comparison of Fatty Acid, Cholesterol and Vitamin Composition in Sea Bass [ *Dicentrarchus labrax* (Linnaeus, 1758)] and Sea Bream [*Sparus aurata* (Linnaeus, 1758)] from Three Cage Farm Areas: Antalya and Muğla (Turkey) and İskele (Northern Cyprus). J. Appl. Ichthyol..

[97] Ozogul Y., Polat A., Uçak İ., Ozogul F. (2011). Seasonal Fat and Fatty Acids Variations of Seven Marine Fish Species from the Mediterranean Sea. Eur. J. Lipid Sci. Technol..

[98] Özyurt G., Polat A., Özkütük S. (2005). Seasonal Changes in the Fatty Acids of Gilthead Sea Bream (*Sparus aurata*) and White Sea Bream (*Diplodus sargus*) Captured in Iskenderun Bay, Eastern Mediterranean Coast of Turkey. Eur. Food Res. Technol..

[99] Costa S., Afonso C., Cardoso C., Oliveira R., Alves F., Nunes M.L., Bandarra N.M. (2016). Towards a Deeper Understanding of Fatty Acid Bioaccessibility and Its Dependence on Culinary Treatment and Lipid Class: A Case Study of Gilthead Seabream (*Sparus aurata*). Br. J. Nutr..

[100] Martínez-Llorens S., Vidal A.T., Moñino A.V., Torres M.P., Cerdá M.J. (2007). Effects of Dietary Soybean Oil Concentration on Growth, Nutrient Utilization and Muscle Fatty Acid Composition of Gilthead Sea Bream (*Sparus aurata* L.). Aquac. Res..

[101] Martinoli M., Contò M., Tonachella N., Cardinaletti G., Martini A., Pulcini D., Renzi G., Failla S., Tibaldi E., Capoccioni F. (2025). Lipid Quality and Oxidative Response in Gilthead Seabream Fillets Fed an Organic Diet Including Crayfish Meal as a Source of Natural Astaxanthin. Meas. Food.

[102] Lomolino G., Crapisi A., Cagnin M. (2016). Study of Elements Concentrations of European Seabass (*Dicentrarchus labrax*) Fillets after Cooking on Steel, Cast Iron, Teflon, Aluminum and Ceramic Pots. Int. J. Gastron. Food Sci..

[103] Žvab Rožič P., Dolenec T., Baždarić B., Karamarko V., Kniewald G., Dolenec M. (2014). Element Levels in Cultured and Wild Sea Bass (*Dicentrarchus labrax*) and Gilthead Sea Bream (*Sparus aurata*) from the Adriatic Sea and Potential Risk Assessment. Environ. Geochem. Health.

[104] Gatta P.P., Pirini M., Testi S., Vignola G., Monetti P.G. (2000). The Influence of Different Levels of Dietary Vitamin E on Sea Bass *Dicentrarchus labrax* Flesh Quality. Aquac. Nutr..

[105] Betancor M.B., MacEwan A., Sprague M., Gong X., Montero D., Han L., Napier J.A., Norambuena F., Izquierdo M., Tocher D.R. (2021). Oil from Transgenic Camelina sativa as a Source of EPA and DHA in Feed for European Sea Bass (*Dicentrarchus labrax* L.). Aquaculture.

[106] Dernekbaşı S., Karayücel İ., Karataş E., Parlak Akyüz A. (2021). Potential of Using Peanut Oil as Alternative to Fish Oil for European Seabass Diets (*Dicentrarchus labrax*) in Recirculated Systems. Alinteri J. Agr. Sci..

[107] Gasco L., Henry M., Piccolo G., Marono S., Gai F., Renna M., Lussiana C., Antonopoulou E., Mola P., Chatzifotis S. (2016). *Tenebrio molitor* Meal in Diets for European Sea Bass (*Dicentrarchus labrax* L.) Juveniles: Growth Performance, Whole Body Composition and in Vivo Apparent Digestibility. Anim. Feed Sci. Tech..

[108] Kocatepe D., Turan H. (2012). Chemical Composition of Cultured Sea Bass (*Dicentrarchus labrax*, Linnaeus 1758) Muscle. J. Food Nutr. Res..

[109] Mastoraki M., Ferrándiz P.M., Vardali S.C., Kontodimas D.C., Kotzamanis Y.P., Gasco L., Chatzifotis S., Antonopoulou E. (2020). A Comparative Study on the Effect of Fish Meal Substitution with Three Different Insect Meals on Growth, Body Composition and Metabolism of European Sea Bass (*Dicentrarchus labrax* L.). Aquaculture.

[110] Baki B., Ozturk D.K., Kerim M. (2019). Comparative Fatty Acıd Composıtıon of European Seabass *Dıcentrarchus labrax* (Linnaeus, 1758) Farmed in Cages in the Aegan Sea and the Black Sea Coasts of Turkey. Indian J. Fish..

[111] Özyurt G., Polat A. (2006). Amino Acid and Fatty Acid Composition of Wild Sea Bass (*Dicentrarchus labrax*): A Seasonal Differentiation. Eur. Food Res. Technol..

[112] Zotos A., Vouzanidou M. (2012). Seasonal Changes in Composition, Fatty Acid, Cholesterol and Mineral Content of Six Highly Commercial Fish Species of Greece. Food Sci. Technol. Int..

